# Estimating Accuracy at Exercise Intensities: A Comparative Study of Self-Monitoring Heart Rate and Physical Activity Wearable Devices

**DOI:** 10.2196/mhealth.7043

**Published:** 2017-03-16

**Authors:** Erin E Dooley, Natalie M Golaszewski, John B Bartholomew

**Affiliations:** ^1^ Department of Kinesiology and Health Education University of Texas at Austin Austin, TX United States

**Keywords:** motor activity, physical exertion, exercise, monitoring, physiologic, energy metabolism, heart rate, photoplethysmography

## Abstract

**Background:**

Physical activity tracking wearable devices have emerged as an increasingly popular method for consumers to assess their daily activity and calories expended. However, whether these wearable devices are valid at different levels of exercise intensity is unknown.

**Objective:**

The objective of this study was to examine heart rate (HR) and energy expenditure (EE) validity of 3 popular wrist-worn activity monitors at different exercise intensities.

**Methods:**

A total of 62 participants (females: 58%, 36/62; nonwhite: 47% [13/62 Hispanic, 8/62 Asian, 7/62 black/ African American, 1/62 other]) wore the Apple Watch, Fitbit Charge HR, and Garmin Forerunner 225. Validity was assessed using 2 criterion devices: HR chest strap and a metabolic cart. Participants completed a 10-minute seated baseline assessment; separate 4-minute stages of light-, moderate-, and vigorous-intensity treadmill exercises; and a 10-minute seated recovery period. Data from devices were compared with each criterion via two-way repeated-measures analysis of variance and Bland-Altman analysis. Differences are expressed in mean absolute percentage error (MAPE).

**Results:**

For the Apple Watch, HR MAPE was between 1.14% and 6.70%. HR was not significantly different at the start (*P*=.78), during baseline (*P*=.76), or vigorous intensity (*P*=.84); lower HR readings were measured during light intensity (*P*=.03), moderate intensity (*P*=.001), and recovery (*P*=.004). EE MAPE was between 14.07% and 210.84%. The device measured higher EE at all stages (*P*<.01). For the Fitbit device, the HR MAPE was between 2.38% and 16.99%. HR was not significantly different at the start (*P*=.67) or during moderate intensity (*P*=.34); lower HR readings were measured during baseline, vigorous intensity, and recovery (*P*<.001) and higher HR during light intensity (*P*<.001). EE MAPE was between 16.85% and 84.98%. The device measured higher EE at baseline (*P*=.003), light intensity (*P*<.001), and moderate intensity (*P*=.001). EE was not significantly different at vigorous (*P*=.70) or recovery (*P*=.10). For Garmin Forerunner 225, HR MAPE was between 7.87% and 24.38%. HR was not significantly different at vigorous intensity (*P*=.35). The device measured higher HR readings at start, baseline, light intensity, moderate intensity (*P*<.001), and recovery (*P*=.04). EE MAPE was between 30.77% and 155.05%. The device measured higher EE at all stages (*P*<.001).

**Conclusions:**

This study provides one of the first validation assessments for the Fitbit Charge HR, Apple Watch, and Garmin Forerunner 225. An advantage and novel approach of the study is the examination of HR and EE at specific physical activity intensities. Establishing validity of wearable devices is of particular interest as these devices are being used in weight loss interventions and could impact findings. Future research should investigate why differences between exercise intensities and the devices exist.

## Introduction

### Prior Research

The *Physical Activity Guidelines for Americans* suggest adults achieve 150 minutes of moderate to vigorous physical activity (MVPA) per week [1]. According to self-report data, the proportion of adults meeting the guidelines was 62.0%, but this dropped to 9.6% for accelerometry measures of activity [2], which illustrates the importance of objective measures of activity. Commercial physical activity tracking wearable devices have emerged as an increasingly popular method for consumers to assess their daily activity and calories expended. In addition to physical activity and energy expenditure (EE), more recent wearable models are designed to measure heart rate at the wrist. In 2015, 232 million wearable electronic devices were sold worldwide, with a projected 18.4% increase in sales for 2016 [3]. With the increase in popularity, wearable-based behavioral change interventions are becoming more prevalent [4-7]. As monitors grow as an intervention tool, questions of validity become paramount and it is important to assess their accuracy for this purpose.

Accuracy is measured by evaluating the device against a research-grade criterion measure. Most research has primarily focused on the accuracy of estimates of EE [7-12]. However, given the pace of technological change, these existing studies have largely been completed on devices that would be considered outdated, such as earlier Fitbit models (One, Zip, Flex, Ultra), Jawbone Up, and the Nike FuelBand. It is important to validate current devices, especially those that are most popular. To this end, our study assesses the Apple Watch, Fitbit Charge HR, and Garmin Forerunner 225. These devices were selected based on review of sales data in 3 categories of wearable devices—smart watches, basic wearable bands, and portable navigation devices [13-16]. However, it should be noted that these devices are not the only commercial devices in the market. These more recent wearable physical activity devices, which include a measure of heart rate, utilize photoplethysmography (PPG) to measure heart rate. PPG is a simple and low-cost optical technique that detects blood volume changes in the microvascular bed of tissue [17]. PPG uses a light source to illuminate the tissue of the wrist and a photodetector to measure variations in light intensity associated with changes in perfusion.

There have been limited validity studies regarding these more recent devices. Fitbit Charge HR has been found to underestimate heart rate and EE [18] with further examination showing the device to have the greatest error in light to moderate physical activity and least amount of error in vigorous physical activity [19]. For Garmin devices, most validation studies focus on the Garmin Vivofit, although Forerunner models have been used in built environment studies. For example, the Forerunner was used to show that, in children, decreased time spent in outdoor environments is associated with increased body weight and lower levels of MVPA [20]. We can find no efforts to validate the Garmin Forerunner 225. Moreover, although the Apple Watch has become very popular, it has only been used in one validation study. The Apple Watch was found to underestimate both heart rate and EE, with a tendency to underestimate calories expended by more than 100 calories [18]. The need for validation is especially important as each of these represent additional technology for behavioral interventions.

To date, most studies analyze accuracy over an entire activity bout in comparison to assessing heart rate accuracy in response to different levels of physical activity intensity. Assessing accuracy in this way may lead to inaccurate results, as over a range of intensities there may be both underestimation and overestimation of feedback. In the few studies that have assessed differences between exercise intensities, results have been mixed depending on device and type of physical activity. Investigators have found that heart rate measurement error increases with activity intensity for the Omron HR-500U and Mio Alpha when jogging, stair climbing, and using the stationary bike [21]. Conversely, another study examining heart rate error during treadmill activities found the highest percentage error in light walking and the least error in running for Mio Alpha, Fitbit Charge HR, Basis Peak, Microsoft Band, and TomTom devices [19]. Therefore, more research is needed to determine the accuracy of these devices in response to various physical activity intensities. Thus, this study will examine 3 popular wearable devices during sedentary behavior, light activity, moderate activity, and vigorous activity to understand the accuracy differences that may occur between intensities.

### This Study

Individual heart rate monitoring during physical activity greatly expands the options for intervention design. Heart rate response to exercise has been shown to be moderated by both knowledge about suggested levels of intensity as well as feedback in meeting those levels [22]. In addition, heart rate feedback has been shown to increase overall daily activity and percentage of time spent in MVPA [23]. Finally, participants who monitored their heart rate following exercise were able to significantly lower their heart rate during recovery, compared with participants who did not have access to monitors [22]. Decreased heart rate recovery rate, or the ability for heart rate to fall rapidly during early recovery after exercise, is associated with increased overall mortality [24]. Thus, if proved accurate, use of heart rate monitors in these devices provides a potentially novel point of intervention during recovery.

Wearable activity monitors are both popular and of increased interest as a component of physical activity interventions. However, little research exists regarding whether these wearable devices—particularly those that measure heart rate—are reliable and valid for these purposes. Thus, the purpose of this study was to compare 3 popular wearable activity monitors with regard to their accuracy in assessing heart rate and estimates of EE at various physical activity intensities. The findings from this study will do much to guide researchers in the selection of wearable activity monitors for future studies’ intervention design.

## Methods

### Participants

A total of 62 students (36 females, 47% nonwhite) aged 18-38 (mean 22.6) years drawn from kinesiology and health courses at a large southwestern university participated in the study. Participants began the testing day after being caffeine-free for 12 hours and having fasted for 3 hours. Those who were current smokers, had a disability that was contraindicated for exercise, or had tattoos, piercings, or braces where the device would be worn were excluded from participation. Participants were compensated with extra credit for their class upon completion of the study. Approval for the study was obtained from the Institutional Review Board at The University of Texas at Austin. Before beginning, all participants provided written informed consent.

### Wearable Devices

Participants simultaneously wore 3 wearable physical activity monitors during testing: Apple Watch, Fitbit Charge HR, and Garmin Forerunner 225. The location of each of the 3 devices (ie, right or left) was randomized before participation.

#### Apple Watch

The Apple Watch (Apple Inc, Cupertino, CA, USA) is an accelerometer-based device that provides estimates of heart rate, distance traveled, calories expended, activity minutes, and standing time. While using the associated Workout app, Apple Watch measures heart rate continuously during a workout, using PPG to calculate beats per minute through green LED lights paired with light-sensitive photodiodes [25]. The heart rate sensor is also designed to compensate for low signal levels by increasing both LED brightness and sampling rate [25]. Calories expended are reported in both active calories and total calories.

#### Fitbit Charge HR

The Fitbit Charge HR (Fitbit Inc, San Francisco, CA, USA) is a triaxial accelerometer–based device that provides estimates of heart rate, steps, calories expended, distance traveled, floors climbed, and sleep quality. The Fitbit Charge HR uses a technology that they label “PurePulse,” which uses LED lights to measure heart rate. Fitbit suggests wearing the device higher on the wrist, 3 finger widths above the wrist bone, to improve accuracy. Calories expended are reported in total calories.

#### Garmin Forerunner 225

The Garmin Forerunner 225 (Garmin, Ltd, Schaffhausen, Switzerland) is an accelerometer-based device that provides estimates of heart rate, steps, calories expended, distance traveled, and sleep time. The device uses GPS (Global Positioning System) and has a built-in optical sensor based on Mio Heart Rate Technology to measure heart rate at the wrist. Mio Heart Rate Technology tracks heart rate using proprietary algorithms for LED light sampling. The frequency at which heart rate is measured varies and depends on the level of activity—as activity increases, the optical heart rate monitor uses a higher sampling rate [26]. Calories expended are reported in total calories.

### Criterion Measures

In addition to the 3 wearable physical activity monitors, participants wore a series of devices to provide criterion measures against which to judge the accuracy of the wearable activity monitors.

#### Polar Heart Rate Monitor

Polar T31 transmitter monitor (Polar Electro, Kempele, Finland) was used as the criterion measure of heart rate as it is a validated and reliable measure for heart rate compared with 12-lead ECG (electrocardiogram) [27]. The heart rate sensor is worn around the chest and transmits real-time heart rate of the user to a wristwatch ECG.

#### Parvo Medics TrueOne 2400

Parvo Medics TrueOne 2400 (Parvo Medics Inc, Sandy, UT, USA) metabolic measurement system was used as the criterion measure for EE in this study. TrueOne 2400 uses a Hans Rudolph pneumotachometer to measure ventilation. EE was estimated from a direct measurement of oxygen consumption and carbon dioxide production. TrueOne 2400 volume and gas were calibrated before each trial. TrueOne 2400 has been previously found to be a reliable measure of EE for research [28].

### Other Measures

#### Physical Activity Intensity

ActiGraph GT3X+ (ActiGraph, Pensacola, FL, USA) accelerometers were used to assess physical activity intensity.

#### Height

A Perspective Enterprises stadiometer (Perspective Enterprises, Portage, MI, USA) was used to measure height to the 0.25 cm. Each participant’s height was measured in workout clothes and without shoes before participation. Height was measured twice, and an optional third measurement was taken if the 2 measurements differed by 0.25 cm. Height was entered into each wearable device and metabolic cart before participation.

#### Weight

Weight was measured using the Tanita BWB-800 scale (Tanita Corporation of America, Arlington Heights, IL, USA). Weight was measured to the nearest 0.1 kg for each participant before participation and the scale was calibrated before each trial. Participants’ weights were measured in workout clothes and without shoes. Weight was measured twice for each participant, and an optional third measurement was taken if the 2 measurements differed by 0.1 kg. Weight was entered into each wearable device and metabolic cart before participation.

#### Ratings of Perceived Exertion

The Borg Rating of Perceived Exertion (RPE) scale was used to measure the participant’s perceived intensity of exercise. The scale value ranges from 6 to 20 and can be used to denote heart rates ranging from 60 to 200 beats per minute [29]. The scale is anchored by no exertion (6), light (11), somewhat hard (13), hard (15), very hard (17), and maximal exertion (20). Participation was suspended if participants indicated they were at maximal exertion.

### Procedures

Participants completed the consent form, a demographic information survey, and were screened for fasting and caffeine consumption before the start of the study. Their participation was rescheduled if the participants did not comply with this criterion. Trained graduate research assistants performed anthropometric measurements. These measurements were used to initialize the wearable devices as well as the metabolic cart for each individual before testing.

To avoid unanticipated problems with device functionality, 2 devices for each product (ie, Fitbit #1 and Fitbit #2) were available for each testing period. The specific device selected (ie, Fitbit #1 or Fitbit #2) and location for each of the 3 devices (ie, right or left arm) were randomized across participants. First, the Polar heart rate strap was placed around the chest and the accelerometer was placed on a belt, positioned on the right hip. Each of the 3 wearable devices was then placed and participants were then fitted with the mouthpiece and nose plug for the metabolic cart. Although accelerometers were used for physical activity intensity validation, this study did not compare EE from this device and therefore will not be used in any analyses.

### Protocol

Initial measures were taken during a 10-minute seated baseline assessment. This was followed by 4 stages of treadmill exercise. Each stage was 4 minutes in length. The final stage was a 10-minute seated recovery period. The activity routine consisted of an unmeasured warm-up walking period and measured stages of walking at 2.5 mph, brisk walking at 3.5 mph, and jogging at 5.5 mph. There was a 1-minute break between each stage. RPE was measured at 1 minute and 3 minutes into each 4-minute stage. To allow sufficient time for participants to reach steady state, heart rate was assessed 3.5 minutes into each stage of activity. These were collected in a random order across devices and averaged together to provide a measure of heart rate for each device during that stage. EE was assessed using the metabolic cart. Activity EE estimates were measured for all stages. Each device was evaluated against the EE estimate of the metabolic cart. Exercise intensity for each participant was evaluated through accelerometry.

### Statistical Analyses

Descriptive statistics were used to examine associations with the criterion measures. Pearson correlations were computed to examine overall group-level associations. Mean absolute percentage error (MAPE) values were calculated as the average absolute value of the errors of each device relative to the criterion measures. Repeated-measures analysis of variance (ANOVA) and Bland-Altman analyses were performed to compare the accuracy of the wearable devices to measure each outcome relative to the criterion measures. Bland-Altman plots were examined for proportional bias. Two-factor ANOVA was performed to compare the effects of sex, body mass index (BMI), and age on the devices. Significant findings were followed with paired-samples *t* tests. Mauchly’s tests were used to test the assumption of sphericity. When violated, degrees of freedom were corrected using Greenhouse-Geisser estimates. Cohen's *d* effect size measures were calculated for each comparison.

## Results

### Participant Characteristics

A total of 62 individuals completed the protocol. Of these, 58% were female (36/62) and 47% were nonwhite (13/62 Hispanic, 8/62 Asian, 7/62 Black/African American, 1/62 other). Ages ranged from 18 to 38 years (mean 22.6 years). BMI ranged from 17.1 to 45.0 (mean 24.6 kg/m^2^). Descriptive statistics for the sample population are provided in Table 1.

**Table 1:** Physical characteristics of participants (N=62).

Characteristics	All (n=62)			Male (n=26)			Female (n=36)		
	Mean	SD	Range	Mean	SD	Range	Mean	SD	Range
Age, years	22.55	4.34	18-38	21.89	2.7	18-29	23.03	5.21	18-38
Height, m	1.70	0.11	1.50-1.92	1.79	0.08	1.58-1.92	1.64	0.09	1.50-1.88
Weight, kg	72.02	18.99	46.36-150.59	85.03	18.77	50.44-150.59	62.63	12.63	46.36-95.62
Body mass index, kg/m^2^	24.60	4.77	17.14-45.02	26.47	5.08	18.36-45.02	23.25	4.1	17.14-33.11

### Heart Rate Overview

The correlations between the criterion scores from the Polar heart rate monitor and the readings from the devices indicate the strongest association with Apple Watch (*r*=.59-.99), followed by the Fitbit Charge HR (*r*=.16-.99), and finally Garmin Forerunner 225 (*r*=.05-.75). Table 2 provides descriptive statistics of heart rate by exercise intensity per device.

**Table 2:** Average heart rate (beats per minute) by exercise intensity per device.

Stage	Device	Mean	SD	Minimum	Maximum	Cohen's *d*
Start	Polar T31	72.32	12.20	46.67	106.00	
	Fitbit Charge HR	73.08	10.44	55.00	102.33	0.07
	Apple Watch	72.84	12.08	43.00	108.00	0.04
	Garmin Forerunner 225	84.90	22.73	58.00	181.33	0.69
Baseline	Polar T31	72.99	11.30	45.00	103.83	
	Fitbit Charge HR	71.36	10.74	47.67	103.33	−0.15
	Apple Watch	73.07	11.45	45.00	105.17	0.01
	Garmin Forerunner 225	80.32	18.38	56.33	169.33	0.48
Light intensity	Polar T31	92.45	13.66	72.00	139.00	
	Fitbit Charge HR	103.11	17.45	73.67	179.33	0.68
	Apple Watch	89.19	11.94	65.00	117.67	−0.25
	Garmin Forerunner 225	108.31	25.69	73.67	166.67	0.77
Moderate intensity	Polar T31	106.84	16.44	78.00	152.00	
	Fitbit Charge HR	110.06	16.71	78.67	162.00	0.19
	Apple Watch	101.01	16.48	68.00	133.00	−0.35
	Garmin Forerunner 225	126.50	23.40	85.33	184.33	0.97
Vigorous intensity	Polar T31	150.63	21.26	112.00	197.67	
	Fitbit Charge HR	144.65	17.35	107.67	192.00	−0.31
	Apple Watch	150.87	19.17	112.00	194.67	0.01
	Garmin Forerunner 225	147.85	21.80	96.67	203.67	−0.13
Recovery	Polar T31	84.47	15.16	46.83	123.17	
	Fitbit Charge HR	82.57	15.17	46.83	119.83	−0.13
	Apple Watch	84.02	15.27	45.17	119.83	−0.03
	Garmin Forerunner 225	87.23	12.48	60.50	120.67	0.20

**Figure 1:**
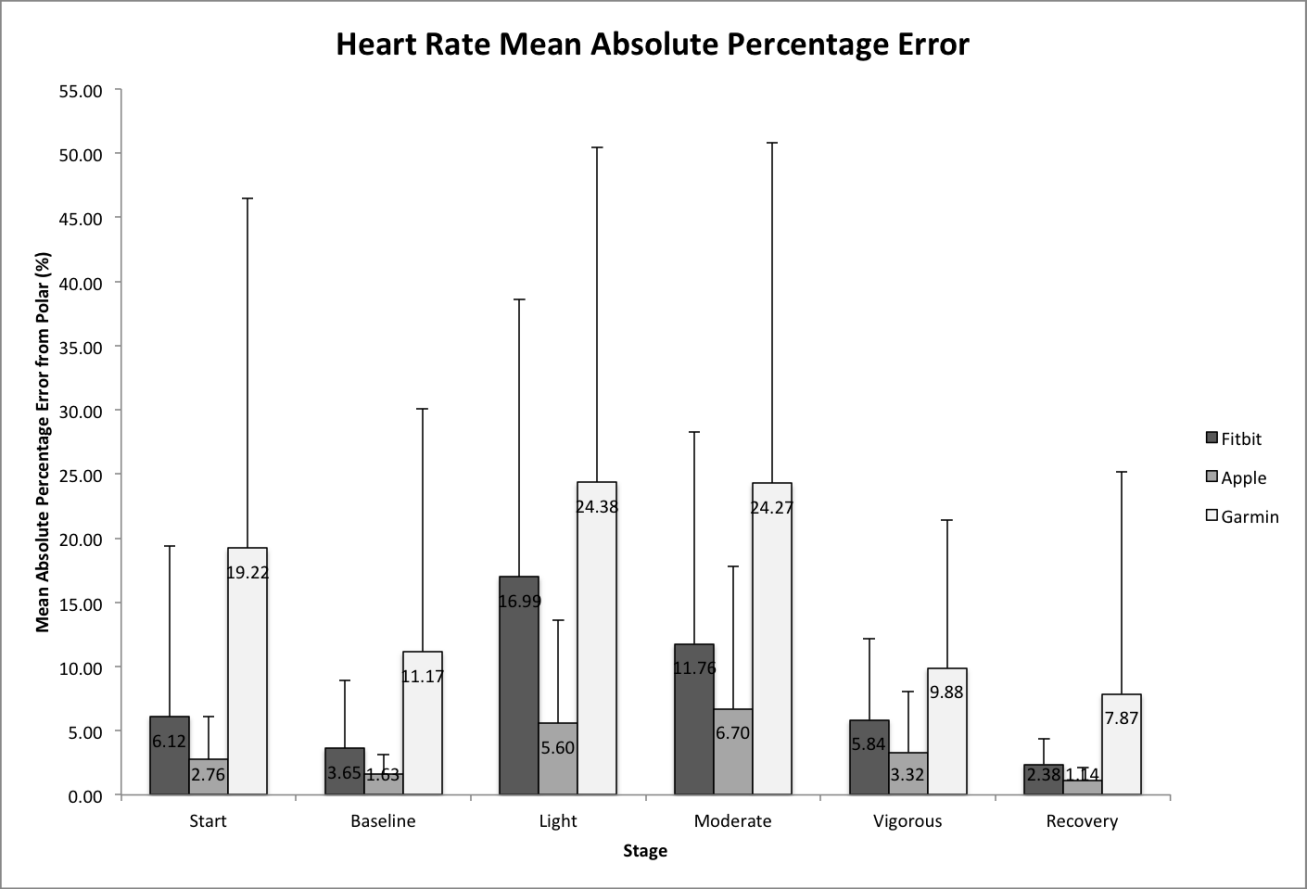
Mean absolute percentage error (MAPE; %) of the devices for heart rate from the Polar heart rate monitor criterion. MAPE values are presented by exercise intensity per device. Error bars represent one standard deviation from the mean score.

Figure 1 shows the MAPE for these devices for heart rate by exercise intensity. The magnitude of errors across all stages was least for the Apple Watch (1.14%-6.70%), followed by the Fitbit Charge HR (2.38%-16.99%) and the Garmin Forerunner 225 (7.87%-24.38%). All repeated-measures ANOVA omnibus *F* tests were significant at the .05 level; therefore, only pairwise comparisons between device (Apple Watch, Fitbit Charge HR, and Garmin Forerunner 225) and criterion measure (Polar heart rate monitor) are reported.

#### Fitbit Charge HR

Fitbit Charge HR was not significantly different from Polar heart rate monitor at the start (*P*=.67) or during moderate intensity (*P*=.34). There was no proportional bias found in Bland-Altman analyses. Fitbit Charge HR measured significantly lower heart rate during baseline (*P*<.001, *d*=0.15), vigorous intensity (*P*<.001, *d*=0.31), and recovery (*P*<.001, *d*=0.13). During light intensity, Fitbit Charge HR measured significantly higher heart rate readings (*P*<.001, *d*=0.68).

#### Apple Watch

Apple Watch was not significantly different from Polar heart rate monitor at the start (*P*=.78), during baseline (*P*=.76), or vigorous intensity (*P*=.84). There was no proportional bias found in Bland-Altman analyses. Apple Watch measured significantly lower heart rate readings during light intensity (*P*=.03, *d*=0.25), moderate intensity (*P*<.001, *d*=0.35), and recovery (*P*=.004, *d*=0.03).

#### Garmin Forerunner 225

Garmin Forerunner 225 was not significantly different from Polar heart rate monitor during vigorous intensity (*P*=.35). There was no proportional bias found in Bland-Altman analysis. Garmin Forerunner 225 measured significantly higher heart rate readings at the start (*P*<.001, *d*=0.69) and during baseline (*P*<.001, *d*=0.48), light intensity (*P*<.001, *d*=0.77), moderate intensity (*P*<.001, *d*=0.97), and recovery (*P*=.04, *d*=0.20).

### Energy Expenditure Overview

The correlations between the criterion scores from Parvo Medics TrueOne 2400 and the readings from the devices indicate the strongest association with Apple Watch (*r*=.59-.87), followed by the Fitbit Charge HR (*r*=.42-.66), and finally Garmin Forerunner 225 (*r*=.18-.73). Table 3 provides descriptive statistics of EE by exercise intensity per device.

**Table 3:** Average energy expenditure (kcal) by exercise intensity per device.

Stage	Device	Mean	SD	Minimum	Maximum	Cohen's *d*
Baseline	Metabolic cart	12.97	3.02	9	25	
	Fitbit Charge HR	11.80	2.79	8	19	−0.40
	Apple Watch	40.41	16.82	17	103	2.27
	Garmin Forerunner 225	32.36	19.27	8	126	1.41
Light intensity	Metabolic cart	14.44	4.14	9	32	
	Fitbit Charge HR	26.32	7.52	15	45	1.96
	Apple Watch	17.15	6.08	9	39	0.52
	Garmin Forerunner 225	24.97	12.98	8	73	1.09
Moderate intensity	Metabolic cart	19.62	4.98	12	32	
	Fitbit Charge HR	27.9	7.68	13	46	1.28
	Apple Watch	21.49	7.24	10	48	0.30
	Garmin Forerunner 225	33.75	10.80	16	69	2.27
Vigorous intensity	Metabolic cart	37.28	9.75	22	64	
	Fitbit Charge HR	38.34	13.63	20	88	0.09
	Apple Watch	40.35	12.51	23	81	0.27
	Garmin Forerunner 225	47.23	16.36	16	92	0.74
Recovery	Metabolic cart	16.61	4.27	10	28	
	Fitbit Charge HR	19.76	12.99	8	64	0.33
	Apple Watch	43.03	19.92	17	105	1.83
	Garmin Forerunner 225	42.65	20.59	13	84	1.75

**Figure 2:**
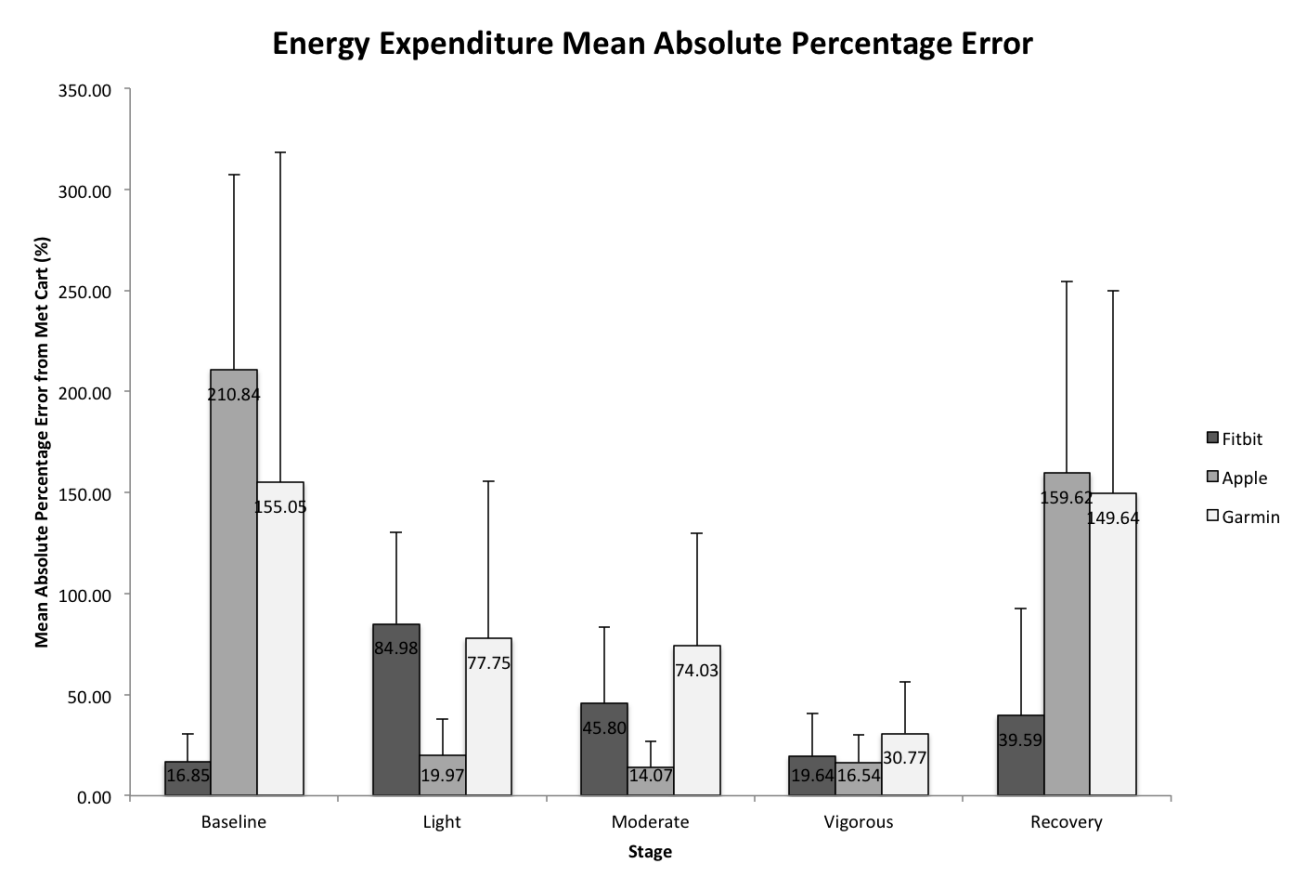
Mean absolute percentage error (MAPE; %) of the devices for energy expenditure from the TrueOne 2400 metabolic cart criterion. MAPE values are presented by exercise intensity per device. Error bars represent one standard deviation from the mean score.

Figure 2 shows the MAPE for these devices for EE by exercise intensity. The magnitude of errors across all stages was least for the Fitbit Charge HR (16.85%-84.98%), followed by the Apple Watch (14.07%-210.84%), and the Garmin Forerunner 225 (30.77%-155.05%). All repeated-measures ANOVA omnibus *F* tests were significant at the .05 level; therefore, only pairwise comparisons between device (Apple Watch, Fitbit Charge HR, and Garmin Forerunner 225) and criterion measure (Parvo Medics TrueOne 2400) are reported.

#### Fitbit Charge HR

Fitbit Charge HR was not significantly different than Parvo Medics TrueOne 2400 during vigorous intensity (*P*=.70) or recovery (*P*=.10). However, Bland-Altman analysis revealed there was bias for overestimation. Fitbit Charge HR measured significantly higher EE during baseline (*P*=.003, *d*=0.40), light intensity (*P*<.001, *d*=1.96), and moderate intensity (*P*<.001, *d*=1.28).

#### Apple Watch

Apple Watch measured significantly higher EE than the criterion measure (Parvo Medics TrueOne 2400) during baseline (*P*<.001, *d*=2.27), light intensity (*P*<.001, *d*=0.52), moderate intensity (*P*<.001, *d*=0.30), vigorous intensity (*P*<.01, *d*=0.27), and recovery (*P*<.001, *d*=1.83). The two-factor ANOVA showed a significant interaction effect of sex and device at baseline, *F*_1,47_=16.74, *P*<.001. The device measured higher EE for males, *t*_24_=12.63, *P*<.001, and females, *t*_33_=10.64, *P*<.001, as compared with the Parvo Medics TrueOne 2400. However, the magnitude of the effect is greater for males (*d*=2.46) than females (*d*=1.6). The two-factor ANOVA showed a significant interaction effect of BMI and device at baseline *F*_3,47_=9.08, *P*<.001. Paired-samples *t* test indicated all BMI categories were significantly different than the criterion. However, the magnitude of the effect is greater for those who were classified as overweight (*d*=3.05), followed by obese (*d*=2.95) and normal weight (*d*=2.92). During moderate intensity, the two-factor ANOVA showed a significant interaction for BMI and device for Apple Watch, *F*_3,48_=8.57, *P*<.001. Paired-samples *t* test determined that overweight and obese BMI categories were significantly different from the criterion; however, normal weight was not significantly different (*P*=.96). The magnitude of the effect is greater for obese (*d*=0.99) than overweight (*d*=0.57) category. During recovery, the two-factor ANOVA revealed a significant interaction for sex and device for the Apple Watch, *F*_1,49_=4.96, *P*<.05; both males, *t*_23_=10.32, *P*<.001, and females, *t*_33_=7.3, *P*<.001, measured higher EE than the criterion. However, the magnitude of the effect is greater for males (*d*=2.58) than females (*d*=1.7). The two-factor ANOVA also revealed a significant interaction for BMI and device for the Apple Watch, *F*_3,49_=8.01, *P*<.001; paired-samples *t* test indicated that all BMI categories were significantly different from the criterion. However, the magnitude of the effect is greater for overweight (*d*=2.60), followed by obese (*d*=2.55) and normal weight (*d*=2.26) categories.

#### Garmin Forerunner 225

Garmin Forerunner 225 measured significantly higher EE than Parvo Medics TrueOne 2400 during baseline (*P*<.001, *d*=1.41), light intensity (*P*<.001, *d*=1.09), moderate intensity (*P*<.001, *d*=2.27), vigorous intensity (*P*<.001, *d*=0.74), and recovery (*P*<.001, *d*=1.75).

## Discussion

### Principal Findings

This study investigated the accuracy of consumer-grade activity monitors for estimating heart rate and EE during stages of sedentary, light, moderate, and vigorous physical activity. Similar to previous heart rate research [19], this study found the highest measurement error for all devices (Apple Watch, Fitbit Charge HR, Garmin Forerunner 225) in light and moderate physical activity stages. The Apple Watch provided the most accurate measure of heart rate relative to the criterion Polar heart rate monitor, as MAPE was between 1.14% and 6.70% for all stages. Fitbit Charge HR showed reasonable results with MAPE between 2.38% and 16.99%. The Garmin Forerunner 225 was the least accurate of the wearable devices, with MAPE between 7.87% and 24.38%. The results indicate the most favorable outcomes for the Apple Watch; however, it measured significantly lower heart rate than the criterion measure during light and moderate physical activity. Fitbit Charge HR produced reasonably accurate results during moderate physical activity but measured significantly higher heart rate readings during baseline and light physical activity and lower heart rate readings during vigorous physical activity. Garmin Forerunner 225 read accurately during vigorous physical activity but measured significantly higher heart rate readings at all other intensities. Results of the Fitbit Charge HR are similar to previous research, which found the highest percentage error in moderate-intensity treadmill activity and the lowest percentage error during vigorous activity [19].

The performance of the devices to measure EE was not accurate either. Apple Watch and Garmin Forerunner 225 report considerably more calories expended than the Parvo Medics TrueOne 2400 metabolic measurement system, which was used as the criterion measure. Fitbit Charge HR measured significantly higher EE for all stages except in vigorous physical activity and recovery. One previous study [10] found MAPE for Fitbit Zip (10.1%) and Fitbit One (10.4%) to be much lower than the results from this study, where MAPE was between 16.85% and 84.98%. Apple Watch has been previously found to underestimate calories by more than 100 calories [18], whereas our study found the device to overestimate calories expended. The differences in results could be due to the previous studies analyzing overall EE and not reporting EE at specific bouts of physical activity intensities. In regard to specific exercise intensity, this study supports previous research that found the Fitbit device to overestimate EE during moderate activity [8]. In our study, the error for EE was the highest for Fitbit Charge HR at light and moderate physical activity.

There are a number of factors that might impact accuracy, such as design, materials, and engineering specifications. Proper placement for optimal sampling from most devices is weak or unknown. Fitbit specified placement higher on the wrist, but there were no instructions for other devices. It may be that varying the location of placement may serve to improve accuracy. Differences in devices in the sampling rate of PPG may also be a cause of variation. Garmin reports varying sampling rate with physical activity intensity [26], but this did not serve to enhance accuracy relative to the other devices. Additionally, the specific algorithms to determine EE for each device are not provided. The increased error from previous studies for the Fitbit device [8,10] suggests that the Fitbit Charge HR may utilize heart rate in determining EE. Errors in heart rate readings would therefore contribute to errors in EE accuracy. The devices are initialized through user’s age, height, and weight measurements. The lack of further anthropometric measures (ie, body fat percentage, waist to hip ratio) to aid in EE assessment could lead to increased errors in estimation. Future research should examine whether additional anthropometric measurements reduce the error in these devices.

### Implications

Behavioral interventions utilizing Fitbit devices have found increased step count and daily physical activity minutes in both adults [5,30,31] and older adults [32]. Likewise, heart rate feedback has been found to increase overall activity and percentage of time spent being vigorously active [23]. Thus, the addition of heart rate monitoring allows further opportunities for researchers to utilize these devices for interventions to increase physical activity and physical activity intensity. However, these data would suggest that, although these are useful as a stimulus to increase activity, each device is limited as an outcome measure or indicator of change in physical activity. As such, researchers would do well to continue to utilize accelerometers or similar well-validated devices for measures of physical activity. The inaccuracy with estimates of EE may be more problematic. Self-monitoring of EE is significantly associated with weight loss and increased daily exercise [33]. However, it is less clear whether wearable physical activity devices aid in weight loss. For example, in one weight loss intervention study, there were no changes in weight for those participants who wore a wearable physical activity device and tracked intake through a food tracking website compared with participants who completed MVPA and food diaries [6]. It could be possible that the wearable devices reported inaccurate EE and consequently participants were consuming more calories than recommended for weight loss. Therefore, questions remain about how the accuracy of these trackers impact interventions. Thus, users and researchers need to be aware of the measurement error for EE within these devices.

To our knowledge, this is the first study to establish the validity of popular wearable devices at distinct physical activity intensities. Most studies establishing validity only assess the overall physical activity intensity for an entire bout of physical activity [18]. Thus, it is difficult to tease apart the differences in heart rate and EE from each wearable device at specific physical activity intensities. Additionally, establishing validity over an entire bout at various physical activity intensities may overestimate accuracy, as the wearable devices may overestimate or underestimate the heart rate and EE at different intensities. In our study, Fitbit Charge HR underestimated heart rate at resting and vigorous intensities but overestimated heart rate at light and moderate intensities. If examined overall, these differences would cancel each other and show minimal differences. Therefore, establishing validity at specific physical activity intensities increases accuracy that we would lose if examined as an overall bout of activity.

Inclusion of specific physical activity intensities is also an advantage as most physical activity or exercise bouts typically stay within certain physical activity intensities. For example, light-intensity activities are of particular importance as older adults tend to spend a greater portion of their day performing at this physical activity level [12], with walking as the most prevalent activity reported among all sociodemographic statuses [34]. Following walking, participation in aerobics is most prevalent in younger women and running most prevalent among younger men [34]. Thus, it is critical to evaluate these devices across different intensities. Unfortunately, this analysis did not find these devices to accurately measure heart rate and EE during light-intensity walking.

### Limitations

There are limitations to this study. This study was conducted in a laboratory setting and therefore may not be generalizable to free-living activities. However, it is necessary to establish validity in a controlled setting, such as in a laboratory, in order to compare potential confounders that might be experienced in a field setting. This laboratory-based study was the necessary first step in device validation against each criterion. Given the observed heart rate variability between the Garmin Forerunner 225 relative to the Polar heart rate monitor, it is probably unnecessary to test in field-based studies, as it does not meet the standards of the criterion in highly controlled settings. However, because of the relative accuracy with heart rate, it is reasonable to suggest further field-testing with the Apple Watch and Fitbit Charge HR for heart rate.

Another limitation is the use of Apple Watch’s EE activity tracking. Using the Workout app, all stages in this study were tracked under “indoor run.” Operating indoor run could be a reason the Apple Watch measured significantly higher EE at baseline and recovery stages. Other activities included in the Workout app are, but not limited to, indoor walk, outdoor walk, elliptical, and other. The differences in algorithms for obtaining EE under various activities may lead to varying EE estimates. However, in order to decrease variability between participants within the study and given that there are no other ways to track EE than operating the Workout app, indoor run was utilized. The Apple Watch also reports both active and total calories per activity. We used total calories in our statistical analyses and did not use active calories because of the generalizability toward the other devices, which all reported overall calories per stage. Further investigation is needed to determine the impact of the Workout app activities on EE.

The Garmin device’s ability to track workouts is a limitation of the device. According to Garmin Ltd, the Forerunner 225 is a GPS running watch, which suggests that the EE algorithm is based solely on running activities, which may be associated with the overestimation at baseline and recovery stages. However, while the differences were significant, both the Apple Watch and Garmin Forerunner 225 devices recorded little to no distance measured during these sedentary activities. As such, the practical impact is likely minimal.

One final limitation is that companies are constantly introducing new updates of activity tracking wearable devices. However, these changes are often aesthetic, with no modification of the underlying technology. For example, while Fitbit models have changed over time, the basic error rate has been similar across studies and devices [8,10]. This suggests that although the devices used in this study underwent software updating, the effect was likely insignificant.

### Conclusions

Despite these limitations, this was one of the first studies to examine the accuracy of consumer-grade activity tracking wearable devices in regard to heart rate. The study used a novel approach to measure accuracy of these devices for heart rate and EE at specific bouts of physical activity intensities. The results of this study provide consumers, researchers, and clinicians the error measurement of 3 popular consumer brands: Apple Watch, Fitbit Charge HR, and Garmin Forerunner 225. Future research should continue to reflect the existing technology and determine why differences between the devices exist. Interventions targeting physical activity through the use of wearable devices should consider these results when selecting a wearable device as an objective measure of physical activity.

## Abbreviations

ANOVA: analysis of variance
BMI: body mass index
ECG: electrocardiogram
EE: energy expenditure
GPS: Global Positioning System
MAPE: mean absolute percentage error
MVPA: moderate to vigorous physical activity
PPG: photoplethysmography
RPE: Rating of Perceived Exertion
